# Association Between Inflammatory Cytokines and Systemic Inflammation Indices in Patients With Psoriasis: A Cross‐Sectional Study

**DOI:** 10.1002/hsr2.71966

**Published:** 2026-03-04

**Authors:** Luca Schneller‐Pavelescu, Maria‐José Sánchez‐Pujol, Esther Caparros‐Cayuela, Rubén Francés‐Guarinos, José‐Manuel Ramos‐Rincón, Isabel Belinchón‐Romero

**Affiliations:** ^1^ Department of Dermatology, Marina Baixa Hospital Villajoyosa Alicante Spain; ^2^ Department of Clinical Medicine Miguel Hernández University Alicante Spain; ^3^ Department of Internal Medicine, Dr. Balmis General University Hospital Alicante Institute for Health and Biomedical Research (ISABIAL) Alicante Spain; ^4^ Department of Dermatology, Dr. Balmis General University Hospital Alicante Institute for Health and Biomedical Research (ISABIAL) Alicante Spain

**Keywords:** biomarkers, cytokines, hematologic tests, inflammation, psoriasis

## Abstract

**Background and Objectives:**

Systemic inflammation indices derived from complete blood counts (CBC) are accessible markers of inflammatory burden, but their relationship with circulating cytokines in psoriasis remains unclear. We investigated associations between CBC‐derived indices and serum cytokines in psoriasis.

**Materials and Methods:**

In this cross‐sectional study, we included 28 patients with psoriasis and 23 healthy controls. Serum IL‐17, IL‐22, IL‐23, IL‐31, IL‐33, IL‐36, TNF‐α, TGF‐β, and IFNγ were quantified by ELISA. CBC‐derived indices were computed (neutrophil‐to‐lymphocyte ratio (NLR), platelet‐to‐lymphocyte ratio (PLR), systemic immune‐inflammation index (SII), systemic inflammation response index (SIRI), and pan‐immune‐inflammation value (PIV). Case–control comparisons used the full sample. Cytokine–index associations were evaluated in psoriasis patients using bivariate correlations and multivariate GLM adjusted for age, smoking, and NAFLD. Multiplicity was controlled using Benjamini–Hochberg false discovery rate (BH‐FDR); prespecified sensitivity analyses used log‐transformed cytokines and Spearman correlations.

**Results:**

Psoriasis patients had higher levels of all cytokines (all *p* < 0.001) and higher SIRI versus controls (*p* < 0.001; *q* = 0.005). PIV showed a nominal case–control difference (*p* = 0.022) that did not remain significant after BH‐FDR (*q* = 0.055), while NLR, PLR, and SII did not differ. In adjusted multivariate GLM, TGF‐β showed a global association with the joint set of indices (Pillai's trace = 0.295; *p* = 0.039) that did not survive BH‐FDR (*q* = 0.507) and was attenuated with log‐transformation. Nominal univariate effects for TNF‐α on SIRI (F = 4.600; *p* = 0.039) and PIV (F = 5.660; *p* = 0.023) did not remain significant after BH‐FDR.

**Conclusions:**

SIRI was consistently elevated in psoriasis, whereas PIV showed a nominal difference versus controls. Across exploratory analyses, SIRI and PIV showed the most consistent directional co‐variation with cytokines, but associations were modest. These findings are hypothesis‐generating and support further validation in larger cohorts to determine whether CBC‐derived indices can serve as scalable adjunct markers of inflammatory activity in psoriasis.

**Abbreviations**
BH‐FDR: Benjamini–Hochberg false discovery rate
CBC: complete blood count
CRP: C‐reactive protein
ELISA: enzyme‐linked immunosorbent assay
ESR: erythrocyte sedimentation rate
FDR: false discovery rate
GLM: general linear model
IL: interleukin
IQR: interquartile range
MRI: magnetic resonance imaging
NAFLD: non‐alcoholic fatty liver disease
NLR: neutrophil‐to‐lymphocyte ratio
PASI: psoriasis area severity index
PIV: pan‐immune‐inflammation value
PLR: platelet‐to‐lymphocyte ratio
SD: standard deviation
SII: systemic immune‐inflammation index
SIRI: systemic inflammation response index
TNF‐α: tumor necrosis factor alpha

## 1 Introduction

Psoriasis is a chronic immune‐mediated skin disease with a prevalence of approximately 2% of the population [1]. The interleukin (IL)−23/IL‐17 axis plays a key role in the pathogenesis of this condition. These cytokines jointly promote systemic inflammation, keratinocyte proliferation, and angiogenesis, factors that contribute to the development of psoriatic plaques [2, 3, 4]. However, systemic inflammation also promotes the onset of psoriatic comorbidities such as psoriatic arthritis, cardiovascular disease, metabolic syndrome, or mood disorders, mediated by cytokines such as tumor necrosis factor alpha (TNF‐α), IL‐6, and IL‐23 [5, 6].

This chronic inflammatory state can also be evaluated through surrogate markers such as alterations in white blood cells (neutrophils, lymphocytes, and monocytes) [7]. Since cytokine measurement may be complex in routine clinical practice, systemic inflammation indices were developed based on these hematologic alterations [8]. Among the most widely used markers are the neutrophil‐to‐lymphocyte ratio (NLR), the platelet‐to‐lymphocyte ratio (PLR), the systemic immune‐inflammation index (SII), the systemic inflammation response index (SIRI), and the pan‐immune‐inflammation value (PIV).

In psoriasis, these indices are increased versus healthy controls and, particularly for NLR and SII, correlate positively with clinical severity measured by psoriasis severity area index (PASI), supporting their use as activity and extent markers [8, 9, 10, 11, 12, 13, 14]. SII and SIRI have been linked not only to prevalent psoriasis but also to incident risk in population cohorts, consistent with low‐grade systemic inflammation contributing to pathogenesis rather than merely reflecting disease burden, while PIV has shown promise for severity stratification and outcome prediction [9, 11, 12, 13]. Longitudinal studies report that NLR, PLR, SII and related indices decline after systemic therapy, especially with TNF‐α and IL‐17A inhibitors, supporting their potential utility as low‐cost monitoring readouts [7, 8, 15]. Associations may vary by phenotype (stronger in pustular or erythrodermic psoriasis), and SII often appears as a more robust independent predictor than simpler ratios [11, 12, 13, 16, 17]. Population‐based data further indicate that sustained elevations in these indices confer higher psoriasis risk after adjustment for age, sex, adiposity, and metabolic comorbidity, with frequently non‐linear relationships and potential risk thresholds [11, 12, 16, 18].

Beyond psoriasis, the same indices demonstrate clinical relevance in rheumatoid arthritis: tracking disease activity and discriminating remission versus active disease, with SII/PIV sometimes outperforming CRP. SIRI is also associated with interstitial lung disease and malignancy [19, 20, 21, 22]. In systemic lupus erythematosus, SII and SIRI relate to global activity and lupus nephritis [23], in systemic sclerosis, NLR and PLR link to pulmonary hypertension, interstitial lung disease, and digital ulcers [24]. In inflammatory bowel disease, NLR and PLR track activity, endoscopic response and relapse risk and NLR predicts steroid response [25]. In patients with osteoarthritis, SIRI, NLR and PLR correlate with clinical activity and patient‐reported impact, whilst SIRI plus CRP improves prediction [26]. In multiple sclerosis patients, SII, NLR and PLR rise with magnetic resonance imaging (MRI)‐active lesions [27], and in metabolic diseases such as NAFLD, linear associations for SII and NLR have been found and non‐linear for PLR [28]. Taken together, CBC‐based indices offer low‐cost, widely available readouts that complement conventional biomarkers yet lack disease specificity and can be confounded. Therefore, integrating them with cytokine profiling may help identify which indices most closely reflect biologically meaningful inflammation [29].

Although numerous studies have profiled circulating cytokines in psoriasis and others have evaluated the systemic inflammation indices [9, 10], to our knowledge, no published work has directly quantified how these two layers of inflammation relate to one another. Establishing the correlation and adjusted association between cytokines and indices is clinically useful for at least three reasons. First, biological validation: testing whether readily available indices (NLR, PLR, SII, SIRI, PIV) truly mirror cytokine‐driven pathways such as the IL‐23/IL‐17 axis. Second, clinical translation: identifying which index best tracks the cytokine signal would enable inexpensive monitoring, risk stratification, and treatment response assessment in routine care or resource‐limited settings where multiplex cytokine assays are impractical. Third, methodological clarity: quantifying effect sizes while adjusting for confounders helps determine whether indices capture independent, biologically meaningful information rather than nonspecific inflammatory background. Accordingly, our objective was to investigate whether systemic inflammation indices (NLR, PLR, SII, SIRI, PIV) are associated with serum cytokine levels in patients with psoriasis, using both bivariate correlations and multivariate adjusted models.

## 2 Materials and Methods

### 2.1 Study Design and Participants

A cross‐sectional observational study was conducted between January and April 2021 at Doctor Balmis General University Hospital (Alicante, Spain). Adults (over 18 years) with a clinical diagnosis of psoriasis followed at the Psoriasis Unit of the Dermatology Department were included. Healthy volunteers with no personal or family history of psoriasis or inflammatory/immunomediated diseases were recruited as controls. Exclusion criteria were age under 18 years, pregnancy, active infections, concomitant autoimmune diseases, or systemic immunosuppressive therapy for reasons other than psoriasis. The study was approved by the hospital Research Ethics Committee and conducted in accordance with the Declaration of Helsinki and applicable data‐protection regulations. Written informed consent was obtained from all participants (patients and controls) covering blood sampling and the use of anonymized clinical and laboratory data for research and publication.

### 2.2 Biomarker Assessment: Cytokines

Demographic, clinical, comorbidity, and treatment data were collected. All participants underwent a single blood draw to obtain routine hematologic parameters and a serum sample for cytokine determination. Serum levels of IL‐17, IL‐22, IL‐23, IL‐31, IL‐33, IL‐36, TNF‐α, TGF‐β, and IFNγ were quantified by ELISA using commercial kits (Invitrogen™, Thermo Fisher Scientific, Waltham, MA, USA). All samples were analyzed in triplicate and read using a Sunrise microplate reader (Tecan, Männedorf, Switzerland). Standard curves were generated per plate and fitted with 4‐parameter logistic models; the optical density of the zero standard was subtracted from all standards, controls, and samples before quantification. We calculated intra‐ and inter‐assay coefficients of variation (CV = standard deviation (SD)/mean × 100%), assessed plate‐to‐plate variability, and evaluated potential plate effects by including plate ID as a random intercept and by comparing plate‐adjusted *vs.* unadjusted models. Triplicates with intra‐assay CV > 15% were re‐run; if one well deviated by > 2 SD from the triplicate mean, it was excluded and the remaining duplicate retained. The analytical lower limit of detection (LOD) was 5 pg/mL. Cytokine levels were analyzed both as continuous variables and as categorical variables based on quartile distribution. For the categorical analysis, psoriasis patients were stratified into two groups: those with cytokine values within the first to third quartiles (Q1–Q3, corresponding to the 0–75th percentiles) and those with values in the highest quartile (Q4, > 75th percentile). This stratification allowed for the comparison of clinical and inflammatory parameters between individuals with lower versus elevated cytokine levels. The continuous analysis enabled the assessment of linear associations between serum cytokine concentrations and systemic inflammation indices.

### 2.3 Systemic Inflammation Indices

Systemic inflammation markers (including NLR, PLR, SII, SIRI, and PIV) were calculated from cell counts obtained from the same blood sample. The NLR was calculated by dividing the absolute neutrophil count by the absolute lymphocyte count. The PLR was calculated by dividing the platelet count by the absolute lymphocyte count. The SII was derived from the formula: (platelet count × neutrophil count)/lymphocyte count. The SIRI was calculated as: (neutrophil count × monocyte count)/lymphocyte count. Finally, the PIV was calculated using the formula: (neutrophil count × platelet count × monocyte count)/lymphocyte count. All cell counts were obtained from routine complete blood count (CBC) analysis and expressed in absolute values (×10⁹/L).

Acute‐phase reactants (erythrocyte sedimentation rate (ESR) and C‐reactive protein (CRP)) were intentionally not collected. The pilot protocol pre‐specified a biomarker panel centered on CBC‐derived indices and a cytokine profile to limit multiplicity and preserve power.

### 2.4 Statistical Analysis

Qualitative variables were expressed as absolute frequencies and percentages, while quantitative variables were expressed as mean and SD (for normally distributed data) or as median and interquartile range (for non‐normally distributed data). Distributional assumptions were assessed using normality tests (Kolmogorov–Smirnov) and inspection of histograms/Q–Q plots; parametric or non‐parametric tests were selected accordingly. Parametric (Student's *t*, ANOVA) or non‐parametric (Mann–Whitney *U*, Kruskal–Wallis) tests were used as appropriate. Bivariate associations between cytokines and systemic inflammation indices were explored using Pearson's r and Spearman's ρ (two‐tailed). Case–control comparisons were performed using the full sample (psoriasis patients and healthy controls), whereas cytokine‐stratified (percentile/quartile) analyses and multivariable cytokine–index models were restricted to psoriasis patients. Quartile/percentile analyses were performed in the psoriasis cohort only, because cytokine distributions differed markedly between cases and controls, and inclusion of controls yielded non‐comparable cut‐points. Cytokine stratification (e.g., > 75th percentile or quartiles) was therefore defined using the psoriasis cohort distribution.

The primary analysis used a multivariate general linear model (GLM) with the set of indices (NLR, PLR, SII, SIRI, PIV) as jointly modeled dependent variables, cytokines as continuous covariates of interest, and age, smoking status, and NAFLD as adjustment covariates. Multivariate significance was evaluated with Pillai's trace (robust to covariance heterogeneity); when significant, univariate between‐subjects effects and parameter estimates were examined. Assumptions were checked via Levene's test and residual diagnostics (standardized residuals vs. fitted values and normal Q–Q plots). Because cytokines are typically right‐skewed, prespecified sensitivity analyses using natural log–transformed cytokine concentrations were conducted to assess robustness of model conclusions.

Given the number of hypothesis tests, multiplicity was controlled using the Benjamini–Hochberg false discovery rate (BH‐FDR). FDR adjustment was applied within pre‐specified families of related hypotheses, and *q*‐values are reported in the corresponding tables. Specifically, BH‐FDR was applied within‐table for case–control comparisons of systemic indices in Table 3 (family size *m* = 5), within‐table for cytokine‐stratified percentile/quartile comparisons (Table 4 and Table S1; family size defined by the number of comparisons reported), for the set of multivariate tests in Table 5 (family=predictors tested against the joint outcome set), and for the set of outcome‐specific univariate follow‐up tests in Table 6 (family size *m* = 45 tests; 9 cytokines × 5 indices). Both unadjusted *p*‐values and BH‐FDR‐adjusted *q*‐values are reported; statistical significance was primarily interpreted as *q* < 0.05. Statistical analyses were performed using SPSS version 24 (IBM, USA).

For multivariate GLMs, covariance heterogeneity was assessed using Box's M test, and Pillai's trace was used as the primary statistic. Homoscedasticity was examined with Levene's tests across outcomes, and residual diagnostics were visually inspected for approximate normality and influential observations.

As this was a pilot study, a formal a priori power calculation was not performed. We report an effect‐size sensitivity analysis aligned with the main objective (association testing): with *n* = 51 and two‐sided α = 0.05, ~80% power is expected for correlations of approximately |*r* | ≈0.38 or larger; within the psoriasis subgroup (*n* = 28), ~80% power corresponds to approximately |*r* | ≈0.51 or larger. This effect‐size sensitivity analysis is intended to contextualize detectable association magnitudes rather than to retrospectively confirm significance. Accordingly, multivariable models were interpreted as exploratory and focused on estimating effect directions and magnitudes.

## 3 Results

### 3.1 Baseline Characteristics and Comorbidity Profile of the Study Population

A total of 51 subjects were analyzed: 28 patients with psoriasis and 23 healthy controls. For cytokine‐stratified analyses, psoriasis patients were categorized using within‐cohort cut‐points (≤ 75th vs > 75th percentile and quartiles) to explore associations with systemic inflammation indices and clinical features. Percentile‐ and quartile‐based stratifications were performed within the psoriasis cohort only, because cytokine distributions differed markedly between cases and controls and would yield non‐comparable cut‐points.

In the descriptive analysis, psoriasis patients had a significantly higher median age compared to healthy controls (46.5 vs 33 years; *p* = 0.036). No significant differences were found regarding sex or obesity prevalence. However, active smoking was significantly more common in the psoriasis group (39.3% vs 4.3%; *p* = 0.010), as was the presence of non‐alcoholic fatty liver disease (NAFLD) (17.9% vs 0%; *p* = 0.001). There were also trends toward a higher frequency of dyslipidemia, metabolic syndrome, and diabetes, although these did not reach statistical significance (Table 1). No statistically significant differences were observed in comorbidities or psoriasis phenotype when patients were stratified by cytokine levels (< 75th vs > 75th percentile) (Tables S1 and S2).

**Table 1:** Baseline characteristics of patients with psoriasis and healthy controls.

	Psoriasis	Healthy controls	*p* value^a^
**Epidemiologic and clinical data**			
Age (years), Median (IQR)	46.5 (16.5)	33.0 (27.0)	**0.036**
Male, *n* (%)	16 (57.1)	11 (47.8)	0.507
Obesity	5 (17.9)	6 (26.1)	0.477
Current smoker, *n* (%)	11 (39.3)	1 (4.3)	**0.010**
Blood hypertension, *n* (%)	7 (25.0)	3 (13.0)	0.285
Dyslipidemia, *n* (%)	10 (35.7)	3 (13.0)	0.065
Diabetes, *n* (%)	3 (10.7)	0	0.106
Metabolic syndrome, *n* (%)	9 (32.1)	3 (13.0)	0.08
NAFLD, *n* (%)	5 (17.9)	0	**0.001**

*Note:* Quantitative variables are expressed as median and interquartile range (IQR), or as mean and standard deviation (SD), while qualitative variables are expressed as absolute numbers (percentage). ^a^Comparisons between groups were performed using the Mann–Whitney *U* test for non‐parametric continuous variables, and Fisher's exact test or chi‐square test for categorical variables and *p*‐value < 0.05 was considered statistically significant (in bold).

Abbreviation: NAFLD, Non‐Alcoholic Fatty Liver Disease.

At enrollment, among the 28 patients with psoriasis, only five were receiving active systemic therapy: one on secukinumab, one on ixekizumab, one on risankizumab, one on ustekinumab, and one on methotrexate. Because exposure was sparse and heterogeneous, treatment status was not included as a covariate in adjusted models; results should therefore be interpreted in the context of potential treatment‐related variability.

### 3.2 Comparative Analysis of Cytokine Profiles and Their Association With Systemic Inflammation Indices

Regarding serum cytokine levels, patients with psoriasis had significantly higher values of IL‐17, IL‐23, IL‐22, IL‐31, IL‐33, IL‐36, TNF‐α, TGF‐β, and IFNγ (all *p* < 0.001) compared to controls (Table 2). In addition, the proportion of participants with cytokine levels above the 75th percentile was higher in psoriasis than in controls for all cytokines (Table S3). Among CBC‐derived systemic inflammation indices, SIRI was higher in psoriasis patients than in healthy controls (*p* < 0.001; *q* = 0.005), whereas PIV showed a nominal difference (*p* = 0.022) that did not remain statistically significant after BH‐FDR correction (*q* = 0.055) (Table 3). NLR, PLR, and SII did not differ between groups (Table 3). When indices were dichotomized at the overall 75th percentile, no between‐group differences were observed (Table S4).

**Table 2:** Serum cytokine levels in patients with psoriasis and healthy controls.

	Psoriasis	Healthy controls	*p* value^a^
**Cytokines (pg/mL)**			
IL‐17, Mean (SD)	9.2 (2.8)	3.0 (1.3)	**< 0.001**
IL‐23, Median (IQR)	11.0 (3.5)	2.7 (1.6)	**< 0.001**
IL‐22, Median (IQR)	1.9 (3.0)	1.0 (0.6)	**< 0.001**
IL‐31, Median (IQR)	88.5 (24.0)	37.4 (24.7)	**< 0.001**
IL‐33, Median (IQR)	143.7 (131.0)	30.7 (24.2)	**< 0.001**
IL‐36, Median (IQR)	90.1 (19.4)	21.1 (15.2)	**< 0.001**
TNFa, Median (IQR)	29.6 (15.2)	0.2 (0.2)	**< 0.001**
TGFb, Median (IQR)	90.1 (14.0)	18.1 (12.5)	**< 0.001**
IFNg, Mean (SD)	2.6 (0.8)	0.6 (0.4)	**< 0.001**

*Note:* Quantitative variables are expressed as median and interquartile range (IQR), or as mean and standard deviation (SD), while qualitative variables are expressed as absolute numbers (percentage). ^a^ Comparisons between groups were performed using the Mann–Whitney *U* test for non‐parametric continuous variables, and Fisher's exact test or chi‐square test for categorical variables and a *p*‐value* <* 0.05 was considered statistically significant (in bold). Given that all comparisons yielded *p <* 0.001, BH‐FDR correction would not alter conclusions; therefore, *q*‐values are not shown.

Abbreviations: IFNγ, Interferon gamma; TGF‐β, Transforming Growth Factor beta; TNF‐α, Tumor Necrosis Factor alpha.

**Table 3:** Systemic inflammatory indices in patients with psoriasis and healthy controls.

	Psoriasis	Healthy controls	*p* value^a^	*q* value (BH‐FDR)
**Inflammatory indices**				
SIRI, Median (IQR)	1.1 (0.7)	0.6 (0.5)	**< 0.001**	**0.005**
PIV, Median (IQR)	273.7 (187.8)	152.2 (239.5)	0.022	0.055
NLR, Median (IQR)	1.8 (1.1)	1.8 (1.0)	0.992	0.155
PLR, Mean (SD)	116.5 (45.0)	142.3 (61.5)	0.093	0.992
SII, Median (IQR)	426.6 (321.6)	369.4 (405.3)	0.961	0.992

*Note:* Continuous variables are reported as median (IQR) or mean (SD) as appropriate. ^a^ Group comparisons were performed using the Mann–Whitney *U* test for non‐normally distributed variables and the independent‐samples t test for normally distributed variables. *p*‐values are twotwo‐sided‐sided and unadjusted. *q*‐values were calculated using the Benjamini–Hochberg false discovery rate (BH‐FDR) procedure within this table across the five index comparisons (*m* = 5). Results were primarily interpreted as statistically significant at *q* < 0.05 (shown in bold), while unadjusted *p*‐values are reported for transparency.

Abbreviations: NLR, neutrophil‐to‐lymphocyte ratio; PIV, pan‐immune‐inflammation value; PLR, platelet‐to‐lymphocyte ratio; SII, systemic immune‐inflammation index; SIRI, systemic inflammation response index.

Nominal percentile‐based differences were observed for selected comparisons within the IL‐17/IL‐23 axis. Specifically, IL‐17 levels above the 75th percentile were associated with higher PIV (*p* = 0.041) and SII (*p* = 0.016), and IL‐23 levels above the 75th percentile showed nominal differences for NLR (*p* = 0.050) and SII (*p* = 0.030) (Table 4). However, after accounting for multiple comparisons using BH‐FDR correction, none of the percentile‐ or quartile‐based comparisons remained statistically significant (all *q* ≥ 0.05; Table 4 and Table S5). Accordingly, these stratified analyses are interpreted as exploratory. In descriptive terms, SIRI and PIV tended to show the most consistent gradients across higher cytokine strata, whereas NLR, PLR, and SII showed less consistent patterns (Table S5). Percentile‐ and quartile‐based comparisons were restricted to psoriasis patients to avoid non‐comparable cut‐points driven by the control distribution.

**Table 4:** Distribution of systemic inflammation indices according to cytokine level percentiles (≤ 75th vs > 75th) for selected cytokines of primary biological interest (IL‐17/IL‐23 axis).

	Percentile ≤ 75th (*n* = 16)	Percentile > 75th (*n* = 12)	*p* value	*q* value (BH‐FDR)
**IL‐17**				
SIRI, Median (IQR)	0.9 (0.8)	1.3 (0.7)	0.103	0.531
PIV, Median (IQR)	206.0 (159.0)	351.3 (202.9)	0.041	0.531
NLR, Median (IQR)	1.6 (1.2)	1.9 (0.8)	0.059	0.531
PLR, Mean (SD)	109.3 (52.4)	125.4 (33.6)	0.363	0.866
SII, Median (IQR)	329.8 (239.2)	548.4 (275.6)	0.016	0.531
**IL‐23**				
SIRI, Median (IQR)	0.9 (0.8)	1.3 (0.6)	0.195	0.866
PIV, Median (IQR)	207.5 (181.3)	343.4 (159.2)	0.134	0.861
NLR, Median (IQR)	1.2 (1.1)	1.9 (0.7)	0.050	0.531
PLR, Mean (SD)	110.3 (50.7)	125.5 (35.3)	0.399	0.866
SII, Median (IQR)	350.5 (221.7)	536.7 (141.0)	0.030	0.531

*Note:* Values are expressed as median (interquartile range (IQR)) for non‐normally distributed variables or as mean (standard deviation (SD)) for normally distributed variables. Comparisons between cytokine quartile groups were performed using the Mann–Whitney *U* test for non‐normally distributed variables and Student's *t*‐test for normally distributed variables. *p*‐values are two‐sided. *q*‐values were calculated using the Benjamini–Hochberg false discovery rate (BH‐FDR) procedure across the full set of percentile‐based comparisons (*m =* 45 tests; 9 cytokines × 5 indices) reported in Supplementary Table 5; *Table* *4* displays the IL‐17/IL‐23 subset for readability. Statistical significance was primarily interpreted at *q <* 0.05; unadjusted *p*‐values are reported for transparency. Cytokine percentiles were computed within the psoriasis cohort and applied only to psoriasis patients.

Abbreviations: NLR, Neutrophil‐to‐Lymphocyte Ratio; PLR, Platelet‐to‐Lymphocyte Ratio; SIRI, Systemic Inflammation Response Index; SII, Systemic Immune‐Inflammation Index.

Exploratory correlation analyses in psoriasis patients suggested broadly concordant directions across Pearson and Spearman methods; however, none of the cytokine–index correlations remained significant after BH‐FDR correction across the 45 tests (all *q* ≥ 0.05). Therefore, correlation results are interpreted as hypothesis‐generating (Figure S1).

When assessing associations between inflammatory indices and cytokines as continuous variables in psoriasis patients, exploratory correlation analyses suggested that SIRI and PIV tended to show the most consistent co‐variation with several cytokines, whereas NLR, PLR, and SII showed weaker or less consistent relationships (Figure S1).

### 3.3 Adjusted Multivariable Associations Between Cytokines and Systemic Inflammation Indices

Multivariable multivariate GLM analyses in psoriasis patients showed no statistically significant global associations between individual cytokines and the joint set of systemic inflammation indices after BH‐FDR correction (all *q* > 0.05). In the primary‐scale analysis, TGF‐β showed a nominal association (Pillai's trace = 0.295; *p* = 0.039), but this did not survive FDR adjustment (*q* = 0.507) and was not replicated in sensitivity analyses using log‐transformed cytokines. Accordingly, multivariate findings are interpreted as exploratory. Homoscedasticity was acceptable across outcomes (Levene's tests *p* > 0.20 for all) (Table 5).

**Table 5:** Multivariate GLM summary for cytokines and covariates.

	Pillai's trace	*F*	*p* (two‐sided)	Partial η²	*q* (BH‐FDR)
IL‐17	0.021	0.135	0.983	0.021	0.983
IL‐23	0.084	0.587	0.71	0.084	0.924
IL‐22	0.107	0.769	0.579	0.107	0.924
IL‐31	0.15	1.127	0.366	0.15	0.685
IL‐33	0.172	1.327	0.278	0.172	0.685
IL‐36	0.149	1.122	0.369	0.149	0.685
TNF‐α	0.213	1.734	0.155	0.213	0.672
TGF‐β	0.295	2.684	0.039	0.295	0.507
IFNγ	0.062	0.423	0.829	0.062	0.954
Age	0.173	1.338	0.274	0.173	0.685
Smoking	0.084	0.586	0.711	0.084	0.924
NAFLD	0.051	0.346	0.881	0.051	0.954

Multivariate tests evaluate the global association of each predictor with the joint set of systemic inflammation indices (NLR, PLR, SII, SIRI, PIV). Models include cytokines as continuous variables and are adjusted for age, smoking status, and NAFLD. Reported metrics are Pillai's trace, F, two‐sided p, False Discovery Rate (FDR)–adjusted q (Benjamini–Hochberg (BH‐FDR) procedure applied within the family of multivariate tests shown in this table), and partial η² (effect size; small≈0.01, medium≈0.06, large≈0.14). All tests were conducted with df1 = 5 and df2 = 32. Pillai's trace is presented as the primary statistic due to its robustness to covariance heterogeneity. Results were primarily interpreted as statistically significant when *q* < 0.05; unadjusted *p*‐values are reported for transparency, and effects with *p* < 0.05 but *q* ≥ 0.05 should be considered nominal/exploratory. Abbreviations: IL, interleukin; TNF‐α, tumor necrosis factor‐α; TGF‐β, transforming growth factor‐β; IFNγ, interferon‐γ; NAFLD, non‐alcoholic fatty liver disease.

Univariate follow‐ups (between‐subjects effects) identified nominal associations for TNF‐α with SIRI and PIV; however, these effects did not remain significant after FDR correction (Table 6). Age showed nominal associations with PIV, PLR, and SII (PIV: *p* = 0.038, *q* = 0.994; PLR: *p* = 0.024, *q* = 0.994; SII: *p* = 0.024, *q* = 0.994), but not with SIRI or NLR. No other cytokines showed FDR‐significant univariate effects on individual indices. Overall model fit was largest for PIV (R² = 0.439; adjusted R² = 0.236) and modest/near‐null for the remaining outcomes (SIRI R² = 0.362; NLR R² = 0.216; PLR R² = 0.245; SII R² = 0.263; several with negative adjusted R² given the number of predictors) (Table 6).

**Table 6:** Outcome‐specific univariate effects (partial η², *F*, and *p*) from multivariable GLMs adjusted for age, smoking, and NAFLD.

	NLR	PLR	SII	SIRI	PIV
IL‐17	η²*p* = 0.004	η²*p* = 0.000	η²*p* = 0.000	η²*p* = 0.009	η²*p* = 0.002
	*F* = 0.146	*F* = 0.004	*F* = 0.001	*F* = 0.336	*F* = 0.082
	*p* = 0.704	*p* = 0.951	*p* = 0.973	*p* = 0.566	*p* = 0.776
	*q* = 0.994	*q* = 0.994	*q* = 0.994	*q* = 0.994	*q* = 0.994
IL‐23	η²*p* = 0.016	η²*p* = 0.024	η²*p* = 0.014	η²*p* = 0.020	η²*p* = 0.015
	*F* = 0.570	*F* = 0.903	*F* = 0.528	*F* = 0.719	*F* = 0.532
	*p* = 0.455	*p* = 0.348	*p* = 0.472	*p* = 0.402	*p* = 0.470
	*q* = 0.994	*q* = 0.994	*q* = 0.994	*q* = 0.994	*q* = 0.994
IL‐22	η²*p* = 0.004	η²*p* = 0.000	η²*p* = 0.009	η²*p* = 0.013	Η²*p* = 0.040
	*F* = 0.138	*F* = 0.001	*F* = 0.334	*F* = 0.483	*F* = 1.507
	*p* = 0.712	*p* = 0.971	*p* = 0.567	*p* = 0.492	*p* = 0.228
	*q* = 0.994	*q* = 0.994	*q* = 0.994	*q* = 0.994	*q* = 0.994
IL‐31	η²*p* = 0.005	η²*p* = 0.000	η²*p* = 0.004	η²*p* = 0.000	η²*p* = 0.005
	*F* = 0.168	*F* = 0.005	*F* = 0.143	*F* = 0.000	*F* = 0.180
	*p* = 0.684	*p* = 0.945	*p* = 0.707	*p* = 0.994	*p* = 0.674
	*q* = 0.994	*q* = 0.994	*q* = 0.994	*q* = 0.994	*q* = 0.994
IL‐33	η²*p* = 0.000	η²*p* = 0.000	η²*p* = 0.010	η²*p* = 0.007	η²*p* = 0.050
	*F* = 0.002	*F* = 0.009	*F* = 0.349	*F* = 0.248	*F* = 1.908
	*p* = 0.969	*p* = 0.923	*p* = 0.558	*p* = 0.621	*p* = 0.176
	*q* = 0.994	*q* = 0.994	*q* = 0.994	*q* = 0.994	*q* = 0.994
IL‐36	η²*p* = 0.000	η²*p* = 0.001	η²*p* = 0.000	η²*p* = 0.009	η²*p* = 0.001
	*F* = 0.002	*F* = 0.030	*F* = 0.003	*F* = 0.344	*F* = 0.022
	*p* = 0.965	*p* = 0.863	*p* = 0.959	*p* = 0.561	*p* = 0.882
	*q* = 0.994	*q* = 0.994	*q* = 0.994	*q* = 0.994	*q* = 0.994
TNF‐α	η²*p* = 0.021	η²*p* = 0.001	η²*p* = 0.038	η²*p* = 0.113	η²*p* = 0.136
	*F* = 0.762	*F* = 0.046	*F* = 1.418	*F* = 4.600	*F* = 5.660
	*p* = 0.388	*p* = 0.831	*p* = 0.241	*p* = 0.039	*p* = 0.023
	*q* = 0.994	*q* = 0.994	*q* = 0.994	*q* = 0.878	*q* = 0.878
TGF‐β	η²*p* = 0.000	η²*p* = 0.028	η²*p* = 0.000	η²*p* = 0.000	η²*p* = 0.017
	*F* = 0.002	*F* = 1.027	*F* = 0.001	*F* = 0.016	*F* = 0.618
	*p* = 0.965	*p* = 0.318	*p* = 0.974	*p* = 0.899	*p* = 0.437
	*q* = 0.994	*q* = 0.994	*q* = 0.994	*q* = 0.994	*q* = 0.994
IFNγ	η²*p* = 0.002	η²*p* = 0.003	η²*p* = 0.006	η²*p* = 0.024	η²*p* = 0.035
	*F* = 0.084	*F* = 0.104	*F* = 0.223	*F* = 0.904	*F* = 1.314
	*p* = 0.774	*p* = 0.749	*p* = 0.639	*p* = 0.348	*p* = 0.259
	*q* = 0.994	*q* = 0.994	*q* = 0.994	*q* = 0.994	*q* = 0.994

*Note:* Each cell reports the partial η² (effect size), *F* statistic, two‐sided *p*‐value, and Benjamini–Hochberg false discovery rate (BH‐FDR)–adjusted *q*‐value for the association between the indicated cytokine (modeled as a continuous predictor) and the specified systemic inflammation index (NLR, PLR, SII, SIRI, PIV), from general linear models adjusted for age, smoking status, and NAFLD. To control for multiple testing across the 45 outcome‐specific comparisons (9 cytokines × 5 indices), *q*‐values were calculated using the BH‐FDR procedure within this family of tests. Statistical significance was primarily interpreted at *q* < 0.05; unadjusted *p*‐values are reported for transparency. Abbreviations: IFN‐γ, interferon‐γ; IL, interleukin; TGF‐β, transforming growth factor‐β; TNF‐α, tumor necrosis factor‐α; NAFLD, non‐alcoholic fatty liver disease.

## 4 Discussion

This study suggests that CBC‐derived systemic inflammation indices may partially reflect cytokine activity in psoriasis. These findings support the notion that psoriasis is not solely a cutaneous condition but a systemic disease marked by widespread immune activation that can be reflected in both molecular biomarkers and indirect hematologic indicators. Given the exploratory design, cross‐sectional setting, and modest sample size, these observations should be interpreted cautiously and warrant validation in larger cohorts.

The primary mediators of chronic inflammation in psoriasis include the IL‐23/IL‐17 axis. IL‐17, produced by Th17 cells, promotes neutrophil chemotaxis and infiltration into target tissues [2, 3], while IL‐23, produced by dendritic cells, sustains Th17 responses and plays a central role in psoriatic inflammation [30]. Additional cytokines, such as IL‐6 (associated with disease severity and comorbidity development) [31] and IL‐22 (sustains the inflammatory cascade and keratinocyte proliferation), also contribute to the inflammatory background [32]. Other cytokines, such as IL‐31, IL‐33 (both also implicated in other chronic inflammatory dermatoses like atopic dermatitis) [33, 34], IL‐36 [35], TNF‐α, TGF‐β, and IFNγ have also been implicated in the chronic inflammatory background observed in psoriasis [36, 37, 38]. In parallel, CBC‐derived indices such as NLR, PLR, SII, SIRI, and PIV summarize shifts across neutrophil, lymphocyte, monocyte, and platelet compartments and have been associated with systemic inflammation and cardiometabolic risk in multiple contexts, including psoriasis [16, 39, 40, 41, 42, 43].

Our findings suggest that higher levels of several cytokines (IL‐17, IL‐23, IL‐31, IL‐33, IL‐36, TNF‐α, TGF‐β, and IFNγ) tended to co‐vary with SIRI and PIV. These two indices are not only increased in psoriasis patients compared to controls but also showed a descriptive stepwise pattern across cytokine quartiles. This observation further supports the hypothesis that they may capture aspects of the inflammatory burden of the disease. While these indices have been proposed as markers of subclinical inflammation in other chronic diseases, our data provide preliminary, hypothesis‐generating evidence of their association with key inflammatory cytokines in psoriasis [44, 45, 46].

Consistent with prior reports showing higher SIRI and PIV in psoriasis, their correlation with PASI, and their decrease after effective therapy [9, 10, 11], our data suggest that these indices may co‐vary with selected cytokines in psoriasis. However, because none of the cytokine–index correlations and stratified comparisons remained significant after BH‐FDR correction, these findings should be considered hypothesis‐generating. Rather than supporting clinical decision‐making at this stage, they motivate prospective longitudinal studies to determine whether changes in SIRI and PIV reliably parallel cytokine dynamics and treatment response.

Importantly, the nominal multivariate signal for TGF‐β did not remain significant after FDR correction and was attenuated in log‐transformed sensitivity analyses, underscoring the need for cautious interpretation and independent replication. Overall, the pattern of findings is consistent with the possibility that systemic inflammatory indices may capture, at least in part, cytokine‐linked inflammatory activity relevant to psoriasis biology, while still reflecting a nonspecific inflammatory burden.

From a clinical standpoint, the main implication of our results is a testable hypothesis that SIRI and PIV could serve as scalable adjunct markers in longitudinal monitoring frameworks (particularly for biologic treatments targeting IL‐17 and IL‐23) where prior studies have documented reductions in these indices among responders [47, 48, 49, 50]. In future prospective cohorts with repeated sampling, it will be important to evaluate whether declines in SIRI and PIV track cytokine suppression and clinical improvement, and whether persistently elevated indices identify patients requiring closer assessment. While effect sizes in our cohort were modest, the directionally concordant findings support further investigation of these low‐cost indices as complementary markers alongside molecular readouts and clinical endpoints.

Notably, TGF‐β was the only cytokine showing a nominal small‐to‐moderate global association with the joint set of systemic inflammation indices in our adjusted multivariate models. Because this signal did not survive BH‐FDR correction and was not robust to log‐transformation, it is discussed here as exploratory. This is biologically plausible: TGF‐β exerts pro and anti‐inflammatory actions and modulates the cellular compartments from which these indices are computed: enhancing neutrophil survival and activation, shaping platelet–lymphocyte crosstalk, and influencing lymphocyte function [51, 52, 53, 54]. Consistent with this, serum TGF‐β levels have been linked to platelet counts and PLR in clinical cohorts, and have been related to disease activity and inflammatory markers in chronic inflammatory disorders, including inflammatory bowel disease [55, 56, 57, 58]. Such mechanisms could shift inflammatory indices upward in settings with neutrophil/platelet predominance and relative lymphopenia, whereas contexts favoring anti‐inflammatory effects could attenuate them. Accordingly, TGF‐β is discussed here as a candidate pathway rather than a confirmed driver of CBC‐derived indices.

In our adjusted models, TNF‐α showed nominal outcome‐specific association with SIRI and PIV, aligning with its well‐established role as an upstream amplifier of innate inflammation in psoriasis, but these did not remain significant after BH‐FDR correction. Mechanistically, TNF‐α promotes neutrophil mobilization and survival (via NF‐κB), augments monocyte activation, and enhances platelet–leukocyte interactions, precisely the cellular compartments that load the numerators of SIRI and PIV. Prior clinical studies have linked higher TNF‐α signaling with elevated inflammatory indices and shown that anti‐TNF therapy lowers NLR, PLR and SII in responders, in parallel with improvements in disease activity [59, 60, 61, 62]. Clinically, these observations motivate prospective studies to determine whether SIRI and PIV can complement clinical endpoints and, where available, molecular biomarkers in longitudinal monitoring [63].

To our knowledge, this is the first study to jointly model a cytokine panel with multiple systemic inflammation indices in psoriasis using adjusted multivariate methods. Beyond simple correlations, we fitted multivariate analysis, quantifying independent associations while avoiding information loss from categorizing continuous variables. While effects were modest, these analyses demonstrate the feasibility of integrating CBC‐derived indices (SIRI, PIV) with circulating cytokine patterns in psoriasis. By suggesting that inexpensive indices may partly reflect the cytokine activity, this work helps bridge molecular and routine laboratory biomarker layers and highlights a practical avenue for future research aimed at scalable inflammatory phenotyping in dermatology.

However, limitations must be acknowledged. The inclusion of both treatment‐naïve and actively treated patients may have influenced cytokine levels and CBC‐derived indices, and the cross‐sectional, single‐center design precludes causal inference and may introduce selection bias. The modest sample size (particularly for multivariable modeling) raises the risk of overfitting and limits power to detect small effects; we therefore used a parsimonious adjustment set and interpret the results as exploratory. To strengthen robustness, we complemented primary‐scale models with log‐transformed cytokine sensitivity analyses and non‐parametric (Spearman) correlation checks. Given multiple comparisons, some findings may be unstable despite FDR control and require replication in larger, preferably longitudinal cohorts. Key clinical covariates and biomarkers were not uniformly available (PASI/BSA at sampling, CRP/ESR, IL‐6/adipokines), limiting confounding control and the ability to distinguish psoriasis‐driven immune inflammation from intercurrent inflammatory states. Finally, controls were convenience volunteers and differed from cases in age and smoking status (with NAFLD absent in controls); although analyses adjusted for major imbalances, residual confounding remains possible in this non‐matched design.

Despite these limitations, this pilot study motivates further research by highlighting hypothesis‐generating associations between circulating cytokines and CBC‐derived systemic inflammation indices in psoriasis. In multivariate models, the strongest signal was a nominal association for TGF‐β that did not survive BH‐FDR correction and was attenuated in log‐transformed sensitivity analyses, underscoring the need for replication. Prospective longitudinal cohorts should test whether SIRI and PIV reliably track cytokine‐linked inflammation and clinical response to biologics targeting the IL‐17/IL‐23 (or IL‐12/23) axis. They should also assess whether these indices help anticipate metabolic or cardiovascular comorbidities. Integrating selected cytokines (e.g., TGF‐β, TNF‐α) with CBC‐derived indices into externally validated models may ultimately support scalable risk stratification and longitudinal inflammatory phenotyping, particularly where cytokine assays are not readily available.

## 5 Conclusions

In this pilot cohort, CBC‐derived systemic inflammation indices—particularly SIRI and PIV—showed directionally concordant but overall exploratory associations with circulating cytokines in psoriasis. Associations were modest and did not remain significant after BH‐FDR correction; a nominal multivariate signal for TGF‐β was attenuated in log‐transformed sensitivity analyses, and outcome‐specific TNF‐α associations were also nominal. Therefore, SIRI and PIV should be considered hypothesis‐generating candidates rather than validated surrogate markers of cytokine activity. Larger, preferably longitudinal cohorts are needed to confirm robustness, assess confounding by intercurrent inflammation, and determine whether trajectories of these indices track cytokine dynamics and treatment response.

## Author Contributions

**Luca Schneller‐Pavelescu:** conceptualization, methodology, validation, formal analysis, investigation, resources, data curation, writing – original draft, preparation, writing – review and editing, visualization, project administration, and funding acquisition. **María José Sánchez‐Pujol:** investigation. **Esther Caparrós‐Cayuela:** investigation and resources. **Rubén Francés‐Guarinos:** investigation and resources. **José‐Manuel Ramos‐Rincón:** conceptualization, methodology, validation, formal analysis, investigation, resources, data curation, writing – review and editing, supervision, project administration, and funding acquisition. **Isabel Belinchón‐Romero:** conceptualization, methodology, validation, investigation, resources, writing — review and editing, supervision, project administration, and funding acquisition. Software: not applicable. All authors have read and agreed to the published version of the manuscript.

## Funding

This research received no external funding. Consequently, there was no funder involvement in the study design; in the collection, analysis, or interpretation of data; in the writing of the manuscript; or in the decision to submit the paper for publication.

## Ethics Statement

The study was conducted in accordance with the Declaration of Helsinki and approved by the Institutional Review Board (Ethics Committee for Research with Medicinal Products of the Alicante Institute for Health and Biomedical Research [ISABIAL], associated with Dr. Balmis General University Hospital) (protocol code PI2020‐172, approved on 25 November 2020).

## Consent

Informed consent was obtained from all patients and controls involved in the study. Written informed consent has been obtained from the patient(s) to publish this paper.

## Conflicts of Interest

The authors declare no conflicts of interest.

## Transparency Statement

The lead author, Luca Schneller‐Pavelescu, affirms that this manuscript is an honest, accurate, and transparent account of the study being reported; that no important aspects of the study have been omitted; and that any discrepancies from the study as planned (and, if relevant, registered) have been explained.

## Supporting information


**Supplementary Figure 1:** Correlation between cytokine levels and systemic inflammation indices in patients with psoriasis. **Table S1:** Distribution of clinical comorbidities according to cytokine levels (≤75th vs >75th percentile). **Table S2:** Distribution of psoriasis phenotypes according to cytokine levels (≤ 75th vs > 75th percentile). **Table S3:** Proportion of participants with cytokine levels above the 75th percentile in psoriasis vs. healthy controls. **Table S4:** Proportion of participants with systemic inflammation indices above the 75th percentile in psoriasis vs. healthy controls. **Table S5:** Distribution of systemic inflammation indices across cytokine quartiles in patients with psoriasis.

## Data Availability

The datasets generated and/or analyzed during the current study are not publicly available due to patient privacy and institutional ethics restrictions. De‐identified data (and SPSS syntax used for analyses) are available from the corresponding author upon reasonable request, subject to approval by the Hospital Doctor Balmis Research Ethics Committee and a data use agreement.
