# Supplementary material for: Association Between Inflammatory Cytokines and Systemic Inflammation Indices in Patients With Psoriasis: A Cross‐Sectional Study
Source: Health Sci Rep. 2026 Mar 4;9(3):e71966. doi: 10.1002/hsr2.71966 (PMC12959472; doi:10.1002/hsr2.71966)
Supplement: Supplementary file 1 — Supplementary Figure 1: Correlation between cytokine levels and systemic inflammation indices in patients with psoriasis. Table S1: Distribution of clinical comorbidities according to cytokine levels (≤75th vs >75th percentile). Table S2: Distribution of psoriasis phenotypes according to cytokine levels (≤ 75th vs > 75th percentile). Table S3: Proportion of participants with cytokine levels above the 75th percentile in psoriasis vs. healthy controls. Table S4: Proportion of participants with systemic inflammation indices above the 75th percentile in psoriasis vs. healthy controls. Table S5: Distribution of systemic inflammation indices across cytokine quartiles in patients with psoriasis. [file HSR2-9-e71966-s001.docx]

Appendix A

Supplementary tables

**Table S1.** Distribution of clinical comorbidities according to cytokine levels (▒≤▒75th vs >75th percentile).

|  | Obesity | | Smoker | | Hypertension | | Dyslipidemia | | Diabetes | | Metabolic syndrome | | NFLAD | |
| --- | --- | --- | --- | --- | --- | --- | --- | --- | --- | --- | --- | --- | --- | --- |
|  | **P**▒<▒**75** | **P**▒>▒**75** | **P**▒<▒**75** | **P**▒>▒**75** | **P**▒<▒**75** | **P**▒>▒**75** | **P**▒<▒**75** | **P**▒>▒**75** | **P**▒<▒**75** | **P**▒>▒**75** | **P**▒<▒**75** | **P**▒>▒**75** | **P**▒<▒**75** | **P**▒>▒**75** |
| IL-23 | 3 (18.8) | 2 (16.7) | 7 (43.8) | 4 (33.3) | 2 (12.5) | 5 (41.7) | 5 (31.3) | 5  (41.7) | 2 (12.5) | 1 (8.3) | 6 (37.5) | 3 (25.0) | 3 (18.8) | 2 (16.7) |
| IL-17 | 3 (18.8) | 2 (16.7) | 8 (50.0) | 3 (25.0) | 3 (18.8) | 4 (33.3) | 5 (31.3) | 5  (41.7) | 2 (12.5) | 1 (8.3) | 6 (37.5) | 3 (27.3) | 2 (12.5) | 3 (25.0) |
| IL-22 | 2 (11.8) | 3 (27.3) | 6 (35.3) | 5 (45.5) | 4 (23.5) | 3 (27.3) | 5 (29.4) | 5  (45.5) | 1 (5.9) | 2 (18.2) | 5 (33.3) | 4 (36.4) | 2 (11.8) | 3 (27.3) |
| IL-31 | 3 (18.8) | 2 (16.7) | 7 (43.8) | 4 (33.3) | 4 (23.5) | 3 (27.3) | 6 (37.5) | 4  (33.3) | 0 | 3 (25.0) | 4 (28.6) | 5 (41.7) | 3 (18.8) | 2 (16.7) |
| IL-33 | 3 (18.8) | 2 (16.7) | 6 (35.3) | 5 (45.5) | 5 (31.3) | 2 (16.7) | 4 (25.0) | 6  (50.0) | 0 | 3 (25.0) | 6 (37.5) | 3 (25.0) | 3 (18.8) | 2 (16.7) |
| IL-36 | 3 (18.8) | 2 (16.7) | 6 (37.5) | 5 (41.7) | 2 (12.5) | 5 (41.7) | 3 (18.8) | 7  (58.3) | 0 | 3 (25.0) | 3 (21.4) | 6 (50.0) | 2 (12.5) | 3 (25.0) |
| TNFα | 2 (11.8) | 3 (27.3) | 6 (37.5) | 5 (41.7) | 3 (18.8) | 4 (33.3) | 3 (18.8) | 7  (58.3) | 0 | 3 (25.0) | 5 (35.7) | 4 (33.3) | 3 (18.8) | 2 (16.7) |
| TGFβ | 1 (6.3) | 4 (33.3) | 7 (43.8) | 4 (33.3) | 4 (25.0) | 3 (25.0) | 4 (25.0) | 6  (50.0) | 0 | 3 (25.0) | 3 (21.4) | 6 (50.0) | 2 (12.5) | 3 (25.0) |
| IFNγ | 3 (18.8) | 2 (16.7) | 7 (43.8) | 4 (33.3) | 3 (18.8) | 4 (33.3) | 7 (43.8) | 3  (25.0) | 2 (12.5) | 1 (8.3) | 5 (33.3) | 4 (36.4) | 2 (12.5) | 3 (25.0) |

Abbreviations: NAFLD: Non-Alcoholic Fatty Liver Disease; TNFα: Tumor Necrosis Factor alpha; TGFβ: Transforming Growth Factor beta; IFNγ: Interferon gamma. Values are expressed as absolute numbers (percentage). Percentile groups are defined as ≤75th percentile (lower cytokine levels) and >75th percentile (higher cytokine levels).

**Table S2.** Distribution of psoriasis phenotypes according to cytokine levels (▒≤▒75th vs >75th percentile).

|  | Scalp | | Palm and sole | | Nail | | Genital | | Arthritis | |
| --- | --- | --- | --- | --- | --- | --- | --- | --- | --- | --- |
|  | **P**▒<▒**75** | **P**▒>▒**75** | **P**▒<▒**75** | **P**▒>▒**75** | **P**▒<▒**75** | **P**▒>▒**75** | **P**▒<▒**75** | **P**▒>▒**75** | **P**▒<▒**75** | **P**▒>▒**75** |
| IL-23 | 11 (68.8) | 8 (66.7) | 2 (12.5) | 0 | 4 (25.0) | 4 (33.3) | 6 (37.5) | 3 (25.0) | 6 (37.5) | 3 (25.0) |
| IL-17 | 12 (75.0) | 7 (58.3) | 2 (12.5) | 0 | 4 (25.0) | 4 (33.3) | 5 (31.3) | 4 (33.3) | 6 (37.5) | 3 (25.0) |
| IL-22 | 11 (64.7) | 8 (72.7) | 2 (11.8) | 0 | 4 (23.5) | 4 (36.4) | 6 (35.3) | 3 (27.3) | 3 (17.6) | 6 (54.5) |
| IL-31 | 9 (56.3) | 10 (83.3) | 1 (6.3) | 1 (8.3) | 6 (37.5) | 2 (16.7) | 5 (31.3) | 4 (33.3) | 6 (37.5) | 3 (25.0) |
| IL-33 | 11 (68.8) | 8 (66.7) | 1 (6.3) | 1 (8.3) | 5 (31.3) | 3 (25.0) | 5 (31.3) | 4 (33.3) | 6 (37.5) | 3 (25.0) |
| IL-36 | 11 (68.8) | 8 (66.7) | 2 (12.5) | 0 | 3 (18.8) | 5 (41.7) | 5 (31.3) | 4 (33.3) | 6 (37.5) | 3 (25.0) |
| TNFα | 10 (62.5) | 9 (75.0) | 2 (12.5) | 0 | 4 (25.0) | 4 (33.3) | 6 (37.5) | 3 (25.0) | 6 (37.5) | 3 (25.0) |
| TGFβ | 10 (62.5) | 9 (75.0) | 2 (12.5) | 0 | 4 (25.0) | 4 (33.3) | 3 (18.8) | 6 (50.0) | 6 (37.5) | 3 (25.0) |
| IFNγ | 11 (68.8) | 8 (66.7) | 2 (12.5) | 0 | 6 (37.5) | 2 (16.7) | 7 (43.8) | 2 (16.7) | 3 (18.8) | 6 (50.0) |

Abbreviations: Tumor Necrosis Factor alpha; TGFβ: Transforming Growth Factor beta; IFNγ: Interferon gamma. Values are expressed as absolute numbers (percentage). Percentile groups are defined as ≤75th percentile (lower cytokine levels) and >75th percentile (higher cytokine levels).

**Table S3.** Proportion of participants with cytokine levels above the 75th percentile in psoriasis vs. healthy controls

|  | **Psoriasis** | **Healthy controls** | **p-value^a^** |
| --- | --- | --- | --- |
| **Cytokines (pg/mL)** |  |  |  |
| IL-17▒>▒P75 (11,8), n (%) | 12 (42.9) | 0 | **<0.001** |
| IL-23▒>▒P75 (13,2), n (%) | 12 (42.9) | 0 | **<0.001** |
| IL-22▒>▒P75 (4,1), n (%) | 11 (39.3) | 0 | **<0.001** |
| IL-31▒>▒P75 (96,6), n (%) | 12 (42.9) | 0 | **<0.001** |
| IL-33▒>▒P75 (231,3), n (%) | 12 (42.9) | 0 | **<0.001** |
| IL-36▒>▒P75 (100,1), n (%) | 12 (42.9) | 0 | **<0.001** |
| TNFα >P75 (37,6), n (%) | 12 (42.9) | 0 | **<0.001** |
| TGFβ >P75 (101,6), n (%) | 12 (42.9) | 0 | **<0.001** |
| IFNγ ▒>▒P75 (3,1), n (%) | 12 (42.9) | 0 | **<0.001** |

Values are n (%). Cut-points shown in parentheses (pg/mL) correspond to the 75th percentile (P75) in the overall study sample. p-valueᵃ from two-sided Fisher's exact test comparing groups; no adjustment for multiple comparisons. Cytokines quantified by ELISA. Abbreviations: IL, interleukin; TNFα, tumor necrosis factor-α; TGFβ, transforming growth factor-β; IFNγ, interferon-γ. ᵃTwo-sided Fisher's exact test.

**Table S4.** Proportion of participants with systemic inflammation indices above the 75th percentile in psoriasis vs. healthy controls

|  | **Psoriasis** | **Healthy controls** | **p-value^a^** |
| --- | --- | --- | --- |
| **Inflammatory indices** |  |  |  |
| SIRI▒>▒P75 (1,5), n (%) | 6 (21.4) | 2 (8.7) | 0.269 |
| PIV▒>▒P75 (365,3), n (%) | 6 (21.4) | 4 (17.4) | 1.000 |
| NLR▒>▒P75 (2,3), n (%) | 6 (21.4) | 5 (21.7) | 0.979 |
| PLR▒>▒P75 (149,6), n (%) | 6 (21.4) | 7 (30.4) | 0.463 |
| SII▒>▒P75 (598,7), n (%) | 6 (21.4) | 8 (34.8) | 0.288 |

Values are n (%). Cut-points shown in parentheses correspond to the 75th percentile (P75) in the overall study sample; indices were calculated from same-day complete blood counts. p-valueᵃ from two-sided Fisher's exact test comparing groups; no adjustment for multiple comparisons. Abbreviations: NLR, neutrophil-to-lymphocyte ratio; PLR, platelet-to-lymphocyte ratio; SII, systemic immune-inflammation index; SIRI, systemic inflammation response index; PIV, pan-immune-inflammation. ᵃTwo-sided Fisher's exact test.

**Table S5.** Distribution of systemic inflammation indices across cytokine quartiles in patients with psoriasis.

| IL-17 | Q1 (n▒=▒13) | Q2 (n▒=▒13) | Q3 (n▒=▒13) | Q4 (n▒=▒12) | p | q |
| --- | --- | --- | --- | --- | --- | --- |
| SIRI, Median (IQR) | 0.6 (0.4) | 0.9 (0.7) | 0.9 (0.7) | 1.3 (0.7) | **0.013** | 0.5310 |
| PIV, Median (IQR) | 146.8 (107.1) | 231.9 (329.3) | 197.9 (140.2) | 351.3 (202.9) | **0.015** | 0.5310 |
| NLR, Median (IQR) | 1.8 (1.4) | 1.7 (1.1) | 1.6 (1.0) | 1.9 (0.8) | 0.291 | 0.5310 |
| PLR, Mean (SD) | 124.7 (41.5) | 150.1 (78.4) | 114.6 (53.9) | 125.4 (33.6) | 0.424 | 0.8658 |
| SII, Median (IQR) | 348.9 (337.5) | 484.8 (450.3) | 329.8 (232.2) | 548.4 (275.6) | 0.121 | 0.5310 |
| IL-23 | **Q1 (n**▒=▒**13)** | **Q2 (n**▒=▒**13)** | **Q3 (n**▒=▒**13)** | **Q4 (n**▒=▒**12)** | **p** |  |
| SIRI, Median (IQR) | 0.6 (0.5) | 0.7 (0.6) | 1.0 (0.7) | 1.3 (0.6) | **0.012** | 0.8658 |
| PIV, Median (IQR) | 144.1 (189.2) | 221.7 (317.6) | 206.0 (158.9) | 343.4 (159.2) | **0.044** | 0.8614 |
| NLR, Median (IQR) | 1.8 (1.4) | 1.3 (1.1) | 1.7 (0.9) | 1.9 (0.7) | 0.224 | 0.5310 |
| PLR, Mean (SD) | 123.5 (39.7) | 148.1 (77.1) | 115.8 (53.1) | 125.5 (35.3) | 0.477 | 0.8658 |
| SII, Median (IQR) | 412.5 (413.6) | 319.7 (460.4) | 371.3 (204.2) | 536.7 (141.0) | 0.345 | 0.5310 |
| IL-22 | **Q1 (n**▒=▒**13)** | **Q2 (n**▒=▒**13)** | **Q3 (n**▒=▒**14)** | **Q4 (n**▒=▒**11)** | **p** |  |
| SIRI, Median (IQR) | 0.6 (0.4) | 1.1 (1.4) | 0.9 (0.7) | 1.0 (0.5) | **0.028** | 0.9430 |
| PIV, Median (IQR) | 144.0 (130.7) | 300.1 (357.3) | 241.5 (197.3) | 262.4 (194.5) | **0.047** | 0.8658 |
| NLR, Median (IQR) | 1.7 (0.7) | 2.1 (1.5) | 1.8 (1.1) | 1.7 (1.1) | 0.285 | 0.8712 |
| PLR, Mean (SD) | 117.0 (23.0) | 137.7 (60.4) | 138.2 (76.9) | 119.1 (42.2) | 0.646 | 0.8879 |
| SII, Median (IQR) | 348.9 (233.1) | 515.0 (387.7) | 459.6 (435.9) | 371.9 (380.0) | 0.147 | 0.8658 |
| IL-31 | **Q1 (n**▒=▒**13)** | **Q2 (n**▒=▒**13)** | **Q3 (n**▒=▒**13)** | **Q4 (n**▒=▒**12)** | **p** |  |
| SIRI, Median (IQR) | 0.7 (0.4) | 0.6 (0.6) | 1.3 (0.9) | 1.1 (0.7) | **0.006** | 0.9430 |
| PIV, Median (IQR) | 146.8 (182.9) | 172.0 (302.6) | 273.7 (246.3) | 254.5 (208.6) | 0.125 | 0.8712 |
| NLR, Median (IQR) | 1.8 (1.2) | 1.4 (0.9) | 2.0 (1.4) | 1.8 (0.4) | 0.678 | 0.8882 |
| PLR, Mean (SD) | 120.6 (47.5) | 161.2 (67.2) | 125.4 (51.9) | 107.1 (37.5) | 0.082 | 0.8658 |
| SII, Median (IQR) | 412.5 (391.3) | 328.9 (510.2) | 385.8 (360.9) | 442.2 (296.6) | 0.897 | 0.8712 |
| IL-33 | **Q1 (n**▒=▒**13)** | **Q2 (n**▒=▒**13)** | **Q3 (n**▒=▒**13)** | **Q4 (n**▒=▒**12)** | **p** |  |
| SIRI, Median (IQR) | 0.6 (0.4) | 0.9 (0.7) | 1.0 (1.2) | 1.1 (0.5) | **0.020** | 0.8712 |
| PIV, Median (IQR) | 115.1 (125.8) | 241.5 (160.4) | 278.7 (403.0) | 276.3 (186.4) | **0.026** | 0.8712 |
| NLR, Median (IQR) | 1.8 (1.0) | 1.8 (1.1) | 1.8 (1.6) | 1.7 (0.6) | 0.905 | 0.8658 |
| PLR, Mean (SD) | 131.0 (52.2) | 151.9 (65.5) | 115.4 (52.3) | 112.9 (41.6) | 0.248 | 0.8712 |
| SII, Median (IQR) | 348.9 (359.0) | 426.6 (404.3) | 455.4 (452.2) | 414.9 (310.2) | 0.471 | 0.8658 |
| IL-36 | **Q1 (n**▒=▒**13)** | **Q2 (n**▒=▒**13)** | **Q3 (n**▒=▒**13)** | **Q4 (n**▒=▒**12)** | **p** |  |
| SIRI, Median (IQR) | 0.6 (0.6) | 0.7 (0.4) | 1.4 (0.5) | 0.9 (0.8) | **0.003** | 0.8658 |
| PIV, Median (IQR) | 146.8 (136.3) | 180.3 (264.9) | 317.9 (95.0) | 180.0 (358.0) | **0.025** | 0.8658 |
| NLR, Median (IQR) | 1.8 (1.1) | 1.7 (0.7) | 2.1 (0.7) | 1.6 (0.7) | 0.476 | 0.8658 |
| PLR, Mean (SD) | 139.8 (50.1) | 134.4 (69.9) | 130.9 (54.4) | 106.8 (36.7) | 0.457 | 0.8658 |
| SII, Median (IQR) | 348.9 (373.1) | 369.4 (440.3) | 475.8 (207.9) | 374.5 (467.7) | 0.749 | 0.8712 |
| TNFα | **Q1 (n**▒=▒**13)** | **Q2 (n**▒=▒**13)** | **Q3 (n**▒=▒**13)** | **Q4 (n**▒=▒**12)** | **p** |  |
| SIRI, Median (IQR) | 0.6 (0.3) | 1.0 (0.9) | 1.1 (0.9) | 1.0 (0.6) | **0.018** | 0.8658 |
| PIV, Median (IQR) | 123.8 (106.1) | 334.7 (283.3) | 242.0 (185.2) | 235.8 (195.9) | **0.017** | 0.8658 |
| NLR, Median (IQR) | 1.4 (0.8) | 1.8 (2.2) | 1.7 (1.2) | 1.7 (0.6) | 0.487 | 0.8712 |
| PLR, Mean (SD) | 129.0 (45.9) | 155.6 (78.2) | 107.3 (31.5) | 119.2 (40.9) | 0.141 | 0.8809 |
| SII, Median (IQR) | 338.2 (285.9) | 635.3 (553.0) | 378.9 (252.3) | 434.8 (345.0) | 0.073 | 0.8712 |
| TGFβ | **Q1 (n**▒=▒**13)** | **Q2 (n**▒=▒**13)** | **Q3 (n**▒=▒**13)** | **Q4 (n**▒=▒**12)** | **p** |  |
| SIRI, Median (IQR) | 0.6 (0.3) | 1.0 (0.7) | 0.9 (0.7) | 1.3 (0.8) | **0.001** | 0.8658 |
| PIV, Median (IQR) | 110.9 (88.0) | 330.2 (248.6) | 215.4 (133.9) | 321.8 (330.9) | **0.002** | 0.8658 |
| NLR, Median (IQR) | 1.4 (0.9) | 1.8 (2.5) | 1.6 (1.0) | 1.8 (0.8) | 0.565 | 0.8712 |
| PLR, Mean (SD) | 135.4 (50.7) | 140.4 (69.3) | 125.0 (55.6) | 110.9 (37.2) | 0.555 | 0.8712 |
| SII, Median (IQR) | 316.8 (285.9) | 600.3 (472.1) | 381.8 (247.9) | 474.9 (376.7) | 0.133 | 0.8712 |
| IFNγ | **Q1 (n**▒=▒**13)** | **Q2 (n**▒=▒**13)** | **Q3 (n**▒=▒**13)** | **Q4 (n**▒=▒**12)** | **p** |  |
| SIRI, Median (IQR) | 0.7 (0.4) | 0.4 (0.9) | 1.0 (0.7) | 1.3 (0.9) | **0.035** | 0.8658 |
| PIV, Median (IQR) | 191.8 (172.8) | 83.4 (304.6) | 241.4 (275.0) | 281.2 (273.1) | 0.263 | 0.8712 |
| NLR, Median (IQR) | 1.8 (1.0) | 1.1 (0.9) | 1.7 (1.1) | 1.9 (1.0) | 0.799 | 0.8658 |
| PLR, Mean (SD) | 133.4 (52.5) | 141.1 (68.8) | 121.8 (57.0) | 115.5 (34.9) | 0.654 | 0.9430 |
| SII, Median (IQR) | 369.4 (351.1) | 253.7 (379.1) | 399.3 (467.3) | 438.9 (266.5) | 0.980 | 0.8712 |

Abbreviations: IL: Interleukin; TNFα: Tumor Necrosis Factor alpha; TGFβ: Transforming Growth Factor beta; IFNγ: Interferon gamma; SIRI: Systemic Inflammation Response Index; PIV: Pan-Immune-Inflammation Value; NLR: Neutrophil-to-Lymphocyte Ratio; PLR: Platelet-to-Lymphocyte Ratio; SII: Systemic Immune-Inflammation Index; IQR: Interquartile Range; SD: Standard Deviation. Values are presented as median (IQR) for non-normally distributed variables and as mean (SD) for normally distributed variables. Quartiles (Q1–Q4) refer to increasing serum cytokine levels. Statistical comparisons across quartiles were performed using the Kruskal–Wallis test or one-way ANOVA as appropriate. A p-value▒<▒0.05 was considered statistically significant (in bold).

**Supplementary Figure 1.** Correlation between cytokine levels and systemic inflammation indices in patients with psoriasis

Abbreviations: TNFα: Tumor Necrosis Factor alpha; TGFβ: Transforming Growth Factor beta; IFNγ: Interferon gamma; SIRI: Systemic Inflammation Response Index; NLR: Neutrophil-to-Lymphocyte Ratio; PLR: Platelet-to-Lymphocyte Ratio; SII: Systemic Immune-Inflammation Index. *Spearman correlation coefficient. **Pearson correlation coefficient. Statistically significant correlations (p▒<▒0.05) are highlighted in green, while non-significant correlations are shown in red. Color intensity reflects the strength of the association or the lack thereof*.*
